# Cigarette Smoke Promotes Interleukin-8 Production in Alveolar Macrophages Through the Reactive Oxygen Species/Stromal Interaction Molecule 1/Ca^2+^ Axis

**DOI:** 10.3389/fphys.2021.733650

**Published:** 2021-10-08

**Authors:** Xianying Zhu, Yuan Zhan, Yiya Gu, Qian Huang, Ting Wang, Zhesong Deng, Jungang Xie

**Affiliations:** ^1^Department of Respiratory and Critical Care Medicine, National Clinical Research Center for Respiratory Disease, Key Laboratory of Pulmonary Diseases of Health Ministry, Tongji Hospital, Tongji Medical College, Huazhong University of Science and Technology, Wuhan, China; ^2^Department of Intensive Care Unit, Sun Yat-sen University Cancer Center, State Key Laboratory of Oncology in South China, Collaborative Innovation Center for Cancer Medicine, Guangzhou, China

**Keywords:** oxidative stress, inflammation, stromal interaction molecule 1, macrophage, chronic obstructive pulmonary disease

## Abstract

Chronic obstructive pulmonary disease (COPD), primarily attributed to cigarette smoke (CS), is characterized by multiple pathophysiological changes, including oxidative stress and inflammation. Stromal interaction molecule 1 (STIM1) is a Ca^2+^ sensor that regulates Ca^2+^ entry in different types of cells. The present study aimed to explore the relationship between CS-induced oxidative stress and inflammation, as well as the functional role of STIM1 thereinto. Our results showed that the reactive oxygen species (ROS)/STIM1/Ca^2+^ axis played a critical role in CS-induced secretion of interleukin (IL)-8 in human alveolar macrophages. Specifically, smokers with COPD (SC) showed higher levels of ROS in the lung tissues compared with healthy non-smokers (HN). STIM1 was upregulated in the lung tissues of COPD patients. The expression of STIM1 was positively associated with ROS levels and negatively correlated with pulmonary function. The expression of STIM1 was also increased in the bronchoalveolar lavage fluid (BALF) macrophages of COPD patients and PMA-differentiated THP-1 macrophages stimulated by cigarette smoke extract (CSE). Additionally, CSE-induced upregulation of STIM1 in PMA-differentiated THP-1 macrophages was inhibited by pretreatment with N-acetylcysteine (NAC), a ROS scavenger. Transfection with small interfering RNA (siRNA) targeting STIM1 and pretreatment with NAC alleviated CSE-induced increase in intracellular Ca^2+^ levels and IL-8 expression. Furthermore, pretreatment with SKF-96365 and 2-APB, the inhibitors of Ca^2+^ influx, suppressed CSE-induced secretion of IL-8. In conclusion, our study demonstrates that CSE-induced ROS production may increase the expression of STIM1 in macrophages, which further promotes the release of IL-8 by regulating Ca^2+^ entry. These data suggest that STIM1 may play a crucial role in CSE-induced ROS production and inflammation, and participate in the pathogenesis of COPD.

## Introduction

Chronic obstructive pulmonary disease (COPD) is a major health problem that causes significant mortality and morbidity worldwide, and therefore places a substantial social and economic burden (Soriano et al., 2017). It is characterized by persistent respiratory symptoms, progressive airflow limitation associated with chronic inflammation and lung destruction, and ultimately irreversible lung function decline. Most cases of COPD are attributed to the exposure to noxious particles or gases, such as cigarette smoke (CS; Hogg and Senior, 2002; Mirza et al., 2018). The pathogenesis of COPD is extremely complicated and thus far remains unclear (Mizumura et al., 2014).

Oxidative stress occurs as a result of an imbalance between the production of free radicals and antioxidant defenses, which plays a crucial role in the pathogenesis of COPD (McGuinness and Sapey, 2017). Reactive oxygen species (ROS) is considered one of the most important by-products in oxygen metabolism and produced substantially during CS inhalation, resulting in a shift of balance to the oxidant side in COPD patients (Fischer et al., 2015). Specifically, oxidative stress promotes the release of proinflammatory mediators, including cytokines and peroxidation products of arachidonic acid (i.e., leukotrienes, prostanoids, and isoprostanes) (Khanna et al., 2013), and induce cell injury and apoptosis by activating and phosphorylating kinase cascades and transcription factors (Rahman, 2005). Oxidative stress is also involved in the genetic and epigenetic regulation of signaling pathways related to emphysema and chronic bronchitis phenotypes (Fischer et al., 2015). As oxidative stress plays a key role in the development of COPD, the therapeutic potential of many antioxidant agents has been evaluated, including thiols [e.g., N-acetylcysteine (NAC), carbocysteine] and antioxidant vitamins (e.g., vitamin C, D, and E) (Biswas et al., 2013; Tse et al., 2013). Chronic airway inflammation is recognized as one of the most critical pathophysiological mechanisms contributing to the pathogenesis of COPD, which causes structural alteration, lumen narrowing, and alveolar destruction (Lange et al., 2021). The relationships among CS, ROS, and inflammation have been under investigation (Lin et al., 2010; Tse et al., 2013). However, the role of stromal interaction molecule 1 (STIM1) in the alveolar macrophages of COPD patients has not been identified.

Stromal interaction molecule 1, a Ca^2+^ sensor located in the endoplasmic reticulum (ER), can promote multiple pathological processes, including inflammation and muscle metabolism, partly by regulating Ca^2+^ entry (Zhang et al., 2014; Kassan et al., 2016). A previous study showed that cigarette smoke extract (CSE) upregulated the protein expression of STIM1 in human airway smooth muscle (Wylam et al., 2015). In addition, lipopolysaccharide (LPS) promoted inflammatory cytokine secretion by increasing the protein expression of STIM1 in murine microglial cells and endothelial cells (Gandhirajan et al., 2013; Heo et al., 2015). STIM1 also acts as a ROS sensor to induce Ca^2+^ entry (Heo et al., 2015). However, the role of STIM1 in the alveolar macrophages of COPD patients has not been identified.

We hypothesized that STIM1 may participate in oxidative stress and pulmonary inflammation in alveolar macrophages and therefore contribute to the pathogenesis of COPD. In the current study, we measured the expression of STIM1 in the lung homogenates and bronchoalveolar lavage fluid (BALF) of COPD patients. The ROS levels and STIM1 expression in CSE-exposed THP-1 cells were examined. CSE-induced changes in cytokine secretion and intracellular Ca^2+^ levels were also explored by treating cells with antioxidant NAC and silencing STIM1.

## Materials and Methods

### Subjects

Normal lung specimens were collected from patients who underwent surgical resection for pulmonary lump in Tongji Hospital, Wuhan, China, between 2017 and 2021. BALF samples were intentionally obtained from patients who underwent bronchoscopy in Tongji Hospital between 2015 and 2017. COPD was diagnosed according to the Global Initiative for Chronic Obstructive Lung Disease (GOLD) criteria (Vestbo et al., 2013). Patients with a post-bronchodilator forced expiratory volume in 1 s (FEV1)/forced vital capacity ratio of less than 70% were enrolled. Age- and gender-matched non-smokers and smokers without COPD were also recruited as control subjects. Participants were excluded if they suffered from asthma, severe lung infections, or other obstructive lung diseases. This study was approved by the Ethics Committees of the Tongji Hospital (TJ-IRB 20140415 and TJ-IRB20210346) and written informed consent was obtained from all subjects.

### Preparation of Alveolar Macrophages

Alveolar macrophages in BALF were obtained by performing bronchoscopy following the international guidelines (Meyer et al., 2012). A total of 120 ml sterile isotonic saline solution at 37°C were separated into four aliquots and flushed into the right middle lobe. After each flush, the fluid was aspirated immediately and gently, collected in sterile centrifuge tubes, and kept on ice. Approximately 50% BALF was recovered. Samples were then filtered through a 40-μm cell strainer and centrifuged at 1,000 × *g* for 10 min at 4°C. Cell pellets were resuspended in RPMI-1640 medium containing 20% fetal bovine serum, 200 U/ml penicillin, and 200 ug/ml streptomycin. Slides were prepared by cytocentrifugation at 1,200 rpm for 5 min and then fixed in 4% formaldehyde for immunofluorescence staining. Cells were cultured in 12-well culture plates in a 5% CO_2_ humidified incubator at 37°C for 2 h. Then, non-adherent cells were removed by washing the plates with RPMI-1640 medium, yielding monolayers that contained at least 95% macrophages. Cell morphology was analyzed and proteins were extracted.

### Preparation of Cigarette Smoke Extract

Cigarette smoke extract was prepared by bubbling the smoke from two burning cigarettes (3R4F, University of Kentucky) at a rate of 1 cigarette/5 min to a 50 ml centrifuge tube containing 20 ml of RPMI-1640 medium. The pH was adjusted to 7.4. The solution was then filtered through a 0.22-μm filter to eliminate bacteria.

### Cell Culture

Human-derived THP-1 cells (ATCC^®^ TIB-202) were cultured in RPMI-1640 medium containing 10% heat-inactivated fetal bovine serum and 1% penicillin/streptomycin in a humidified incubator with 5% CO_2_ at 37°C. Cells were treated with phorbol myristate acetate (PMA, 100 nM) for 48 h to induce macrophage differentiation. In transfection experiment, cells were transfected with 50 nM small interfering RNA (siRNA) targeting STIM1 (5′-GTGGTACAGTGGCTGATCA-3′) or negative control sequence (RiboBio, Guangzhou, China) using Lipofectamine 3000 (Invitrogen, Carlsbad, CA, United States) according to the manufacturer’s instructions. In pharmacological experiment, cells were pretreated with 3 mM NAC (Sigma-Aldrich, St. Louis, MO, United States) for 1 h, with 10 μM SKF-96365 (MedChemExpress, United States) for 2 h, or with 10 μM 2-APB (MedChemExpress, United States) for 2 h before CSE stimulation.

### Immunohistochemical Analysis

Formalin (10%)-fixed, paraffin-embedded lung tissue sections of healthy non-smokers (HN), smokers without COPD, and smokers with COPD (SC) were deparaffinized using xylene and rehydrated in a graded ethanol series. Heat-induced antigen retrieval was performed using a microwave. After cooling with running tap water, sections were incubated with 3% hydrogen peroxide to block endogenous peroxidase activity, followed by 1-h incubation in 5% BSA-phosphate-buffered saline (PBS) solution at room temperature to avoid non-specific background. Then, slides were incubated with polyclonal rabbit anti-STIM1 (1:500; Proteintech, United States) antibody at 4°C overnight in a humidified chamber. After washing, sections were incubated with a peroxidase-conjugated goat anti-rabbit secondary antibody for 1 h at room temperature. The reactions were developed using a DAB substrate kit with hematoxylin as a counterstain. A Nikon Spot image acquisition and processing system (United States) was used for image assessment.

### Immunofluorescence Staining

Cells were fixed with 4% paraformaldehyde for 15 min and stored at −80°C. After washing, slides were incubated in 3% hydrogen peroxide solution in the dark for 10 min. After three washes with PBS for 5 min, slides were blocked in 5% BSA for 20 min. Then, slides were incubated with polyclonal rabbit anti-STIM1 antibody (1:50, Proteintech, United States), monoclonal mouse anti-CD68 antibody (Abcam, United Kingdom), or isotype controls (Becton Dickinson, Franklin Lakes, United States) overnight at 4°C. After washing, slides were incubated with goat anti-rabbit and goat anti-mouse secondary antibodies (Aspen, Wuhan, China) for 50 min at 37°C. DAPI was used to counterstain the nuclei.

### Detection of Reactive Oxygen Species Levels

Lung tissues (1 g) were obtained from subjects who underwent surgical resection as aforementioned and flash frozen with liquid nitrogen. Samples were thawed, maintained at 2–8°C, and homogenized with 9 mL PBS by a grinder. Then, tissue homogenates were centrifuged at 3,000 rpm for 10 min and the supernatant was collected and examined for concentration. The supernatants were incubated with 1 mmol/L DCFH-DA solution (Elabscience, China) for 30 min at 37°C. The ROS levels were detected by a microplate reader (Molecular Devices, China) at 525 nm.

The levels of ROS in THP-1 cells were detected by a Fluorometric Intracellular Ros Kit (Sigma-Aldrich, Darmstadt, Germany). After pretreated with NAC for 1 h, PMA-differentiated THP-1 cells were treated with CSE for 3 h. Then, cells were washed and incubated with RPMI-1640 containing 1 μM dichlorofluorescein diacetate (H2DCF-DA) for 30 min. After three washes with RPMI-1640 medium for 5 min, cells were digested by trypsin, washed again, and resuspended in 200 μl PBS. Finally, flow cytometry (BD Biosciences, SanJose, CA, United States) was performed to detect the fluorescent signal intensity at 525 nm to indicate the intracellular levels of ROS.

### Measurement of Intracellular Ca^2+^

Intracellular Ca^2+^ levels were measured using the Fluo-3, AM Kit (Solarbio, Beijing, China) in accordance with the manufacturer’s protocol. After treated with CSE or other indicated agents, cells were incubated with 5 μM molecular probe Fluo-3, AM for 20 min at 37°C. Next, cells were cultured in HBSS containing 1% fetal bovine serum for 40 min and then resuspended in HBSS buffer saline. The Ca^2+^ levels were determined by flow cytometry (BD Biosciences, SanJose, CA, United States) at 525 nm.

### Cell Viability Assay

Approximately 5,000 cells were seeded in each well of a 96-well plate (Corning, MA, United States). After 48-h incubation with different concentrations of CSE, Cell Counting Kit-8 (CCK-8; Promoter Biotechnology, Wuhan, China) was used to detect cell viability according to the manufacturer’s instructions. Optical density (OD) values were obtained using a microplate reader (Molecular Devices, China).

### ELISA

The levels of interleukin (IL)-8 and IL-1β in PMA-differentiated THP-1 macrophages were quantified using DuoSet ELISA kit (R&D Systems, Minneapolis, MN, United States) and RayBio ELISA kit (RayBiotech, United States) according to the manufacturers’ instructions. The minimum detectable dose was 31.3 pg/mL and 0.3 pg/ml for IL-8 and IL-1β, respectively.

### Real-Time Quantitative Polymerase Chain Reaction

Total RNA was extracted using Trizol (Takara, Japan) and reverse transcribed to cDNA using PrimeScript^TM^ RT reagent Kit with gDNA Eraser (Takara, Japan). The mRNA expression was assessed by real-time quantitative polymerase chain reaction (RT-qPCR) using TB Green^®^ Premix Ex Taq^TM^ II (Takara, Japan) on BioRad CFX384 (Bio-Rad, CA, United States). The parameters were as follows: 40 cycles at 95°C for 10 s, 59°C for 20 s, and 72°C for 30 s. The relative mRNA expression was determined using the 2^–^^Δ^^Δ^^Ct^ method with β-actin as the internal control. The following primers were used in this experiment: β-actin (F: 5′-GCGCGGCTACAGCTTCA-3′; R: 5′-CTTAATGTCACGCACGATTTCC-3′), STIM1 (F: 5′-TTG TCCATGCAGTCCCCTAG-3′; R: 5′-GGTAGTGGTGATGGTG GTGA-3′).

### Western Blot

Total protein was extracted using RIPA buffer supplemented with protease inhibitor cocktail (Servicebio, Wuhan, China). Equal amounts of proteins were separated by 10% sodium dodecyl sulfate-polyacrylamide gel electrophoresis (SDS-PAGE) and transferred onto polyvinylidene fluoride (PVDF) membranes. After blocking with Tris-buffered saline and tween-20 (TBST) containing 5% non-fat milk for 1 h, the membranes were incubated with rabbit polyclonal anti-STIM1 antibody (1:1,000, Proteintech, Chicago, IL, United States) or rabbit polyclonal anti-β-tubulin antibody (1:4,000, Sungene biotech, Shanghai, China) overnight at 4°C. The membranes were washed with TBST buffer, followed by 1-h incubation with a horseradish peroxidase-conjugated goat anti-rabbit IgG antibody (1:4,000; Sigma-Aldrich, Darmstadt, Germany) at room temperature. After washed with TBST, the protein expression was detected using an ECL chemiluminescence detection kit (Advansta, California, United States) and quantified by ImageJ.

### Statistical Analysis

All data were normally distributed and expressed as the mean ± SEM. Statistical significance was determined by Student’s *t*-test or one-way ANOVA with Newman–Keuls multiple comparison test as appropriate. The correlations were analyzed by Pearson correlation. The GraphPad Prism 8 Software (GraphPad Software, San Diego, CA, United States) was used for all statistical analysis and graphic generation. The FlowJo 10 software was used for flow cytometry analysis. *P* < 0.05 was considered statistically significant.

## Results

### Characteristics of All Subjects

The clinical characteristics of all subjects are shown in Tables 1, 2. Lung tissues were collected from 12 HN, 12 smokers without COPD (healthy smokers, HS), and 16 SC. BALF samples were obtained from 13 HN, 14 HS, and 12 SC. There was no significant difference in age and gender among these groups. Compare with the HN group, the smoking index of HS and SC was markedly increased. The FEV1% predicted and FEV1/FVC of SC were significantly lower than those of the HN and HS groups.

**TABLE 1:** Clinical characteristics for those subjects with collected lung tissues.

	**HN(*n* = 12)**	**HS(*n* = 12)**	**SC(*n* = 16)**
Age (yrs)	61.5(1.3)	60.3(3.0)	62.7(1.7)
Gender	12/0	12/0	16/0
Smoking (pack-yrs)	0	40.0(7.4)*	41.2(6.0)*
FEV1% predicted	104.1(3.4)	93.1(4.5)	82.7(6.7)*
FEV1/FVC	83.3(2.1)	74.5(2.0)*	60.6(2.5)*^#^

Values are expressed as mean(SEM); HN, healthy non-smoker. HS, healthy smoker. SC, smoker with COPD; FEV1: forced expiratory volume in 1 s; FVC: forced vital capacity; *<0.05 vs. patients in HN group; ^#^<0.05 vs. patients in HS group.

**TABLE 2:** Clinical characteristics for those subjects with collected alveolar macrophages.

	**HN(*n* = 13)**	**HS(*n* = 14)**	**SC(*n* = 12)**
Age (yrs)	54.9(3.0)	55.1(2.6)	59.5(1.6)
Gender	13/0	14/0	12/0
Smoking (pack-yrs)	0	43.9(7.3)*	52.3(8.4)*
FEV1% predicted	102.4(4.2)	96.7(3.8)	64.6(3.9)*^#^
FEV1/FVC	77.2(1.3)	75.9(1.2)	56.5(3.5)*^#^

Values are expressed as mean(SEM); HN, healthy non-smoker. HS, healthy smoker. SC, smoker with COPD; FEV1: forced expiratory volume in 1 s; FVC: forced vital capacity; *<0.05 vs. patients in HN group; ^#^<0.05 vs. patients in HS group.

### Intrapulmonary Expression of Stromal Interaction Molecule 1 Is Increased in Chronic Obstructive Pulmonary Disease Patients

**FIGURE 1:**
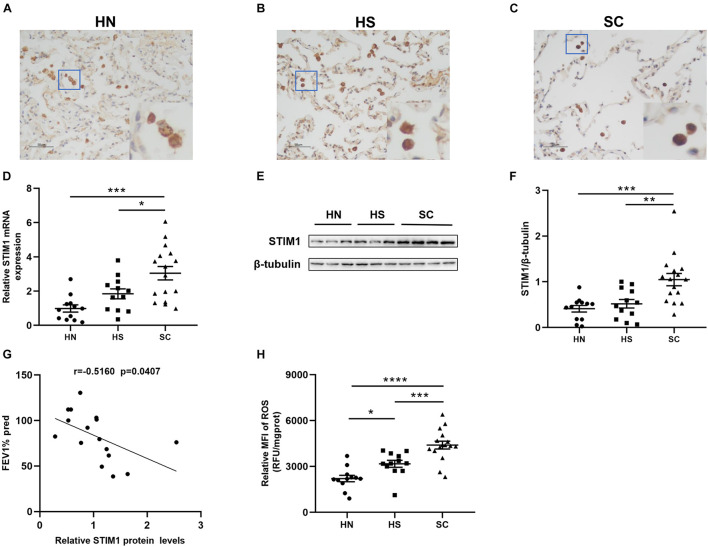
Intrapulmonary stromal interaction molecule 1 (STIM1) expression and reactive oxygen species (ROS) levels are enhanced in the lung of chronic obstructive pulmonary disease (COPD) patients. Representative immunohistochemical images of human lung tissues against STIM1 are shown. Compared with healthy non-smokers (HN) **(A)** and healthy smokers (HS) **(B)**, STIM1 expression was markedly increased in the alveolar macrophages of smokers with COPD (SC) **(C)**. Magnification = × 200. The red-boxed area indicates a region of higher magnification. The STIM1 mRNA expression was increased in SC relative to HN and HS **(D)**. Representative STIM1 expression in whole-lung tissue homogenates was detected by Western blot **(E)** and quantified using ImageJ **(F)**. Increased STIM1 is negatively associated with pulmonary function in COPD patients **(G)**. The ROS levels of lung tissue in SC patients were significantly increased relative to HS and HN **(H)**. Data are displayed as mean ± SEM, *n* = 12 for HN, *n* = 12 for HS, and *n* = 16 for SC. *P*-values were calculated using one-way ANOVA followed by Newman–Keuls test. *P < 0.05, **P < 0.01, ***P < 0.001, and ****P < 0.0001 represent significant differences. STIM1, stromal interaction molecule 1; FEV1%pred, forced expiratory volume in 1 s (FEV1)% predicted; ROS, reactive oxygen species; MFI, Mean fluorescent intensity; HN, healthy non-smoker; HS, healthy smoker; and SC, smoker with COPD.

Immunohistochemistry was performed to detect the expression of STIM1 in the lung tissue of HN, HS, and SC (Figures 1A–C). STIM1 was mainly expressed in alveolar macrophages. Compared with the HN and HS groups, the expression of STIM1 in SC was notably increased. RT-qPCR and Western blot were performed to validate the expression of STIM1. Consistent with the results of immunohistochemistry, intrapulmonary expression of STIM1 in SC was prominently increased compared with the HN and HS group at both mRNA (Figure 1D) and protein (Figures 1E,F) levels. To explore the association between STIM1 expression and pulmonary function, the correlation between STIM1 protein expression and FEV1% predicted in the SC group was analyzed. We found that intrapulmonary STIM1 expression was negatively correlated with the pulmonary function of COPD patients (Figure 1G), indicating the potential role of STIM1 in the development of COPD.

### Increased Reactive Oxygen Species Levels in Smokers With or Without Chronic Obstructive Pulmonary Disease

The levels of ROS in the lung tissue supernatants of SC and HS were significantly increased compared with the HN group (Figure 1H). HS showed higher ROS levels than HN, indicating that CS may directly lead to the elevation of ROS levels in the lungs. Moreover, significantly higher levels of ROS were observed in the lung tissues of SC compared with HS, suggesting enhanced oxidative stress in the lungs of SC. To determine the association between STIM1 expression and ROS production, we performed correlation analysis in all subjects. The results showed that pulmonary ROS levels were positively associated with STIM1 expression at both mRNA (Supplementary Figure 1A) and protein (Supplementary Figure 1B) levels.

### Stromal Interaction Molecule 1 Is Expressed in Human Alveolar Macrophages and Upregulated in Macrophages Isolated From the Bronchoalveolar Lavage Fluid of Chronic Obstructive Pulmonary Disease Patients

**FIGURE 2:**
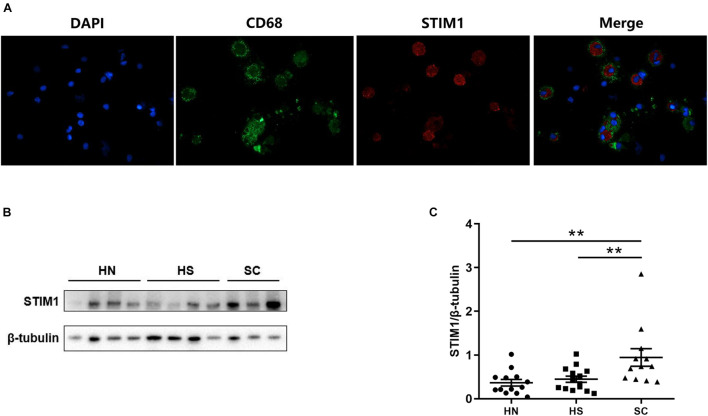
Stromal Interaction Molecule 1 is located in the human alveolar macrophages and up-regulated in macrophages isolated from bronchoalveolar lavage fluid (BALF) of COPD patients. Representative immunofluorescent images against STIM1 in macrophages isolated from BALF are shown **(A)**. Representative western blot images of STIM1 expression in the macrophages of BALF are shown **(B)** and quantified using ImageJ **(C)**. Data are displayed as mean ± SEM, *n* = 13 for HN, *n* = 14 for HS, and *n* = 12 for SC. *P*-values were calculated using one-way ANOVA followed by Newman–Keuls test. **P < 0.01 represent significant differences. STIM1, stromal interaction molecule 1; BALF: bronchoalveolar lavage fluid; HN, healthy non-smoker; HS, healthy smoker; and SC, smoker with COPD.

Previous immunohistochemistry analysis showed that STIM1 was mainly expressed in alveolar macrophages. To further define the location and expression level of STIM1, immunofluorescence staining and Western blot were performed using alveolar macrophages isolated from the BALF samples. Immunofluorescence staining against CD68 (a marker of macrophages), STIM1, and DAPI revealed that STIM1 was predominantly located in the cytoplasm rather than the nucleus of macrophages (Figure 2A). Moreover, alveolar macrophages from different individuals were cultured for 24 h and the protein expression of STIM1 was detected by Western blot. The level of STIM1 in SC was significantly higher than that of the HS and HN groups, and there was no significant difference between the HN and HS groups (Figures 2B,C). These results indicated that STIM1 may play a key role in the development of COPD. We therewith treated human macrophages with 5% CSE to investigate the effect of CSE on STIM1 expression. We found that CSE significantly upregulated the expression of STIM1 in alveolar macrophages *in vitro* (Supplementary Figures 2A,B).

### N-Acetylcysteine Alleviates Cigarette Smoke Extract-Induced Reactive Oxygen Species Production and Stromal Interaction Molecule 1 Expression in Phorbol Myristate Acetate-Differentiated THP-1 Cells

**FIGURE 3:**
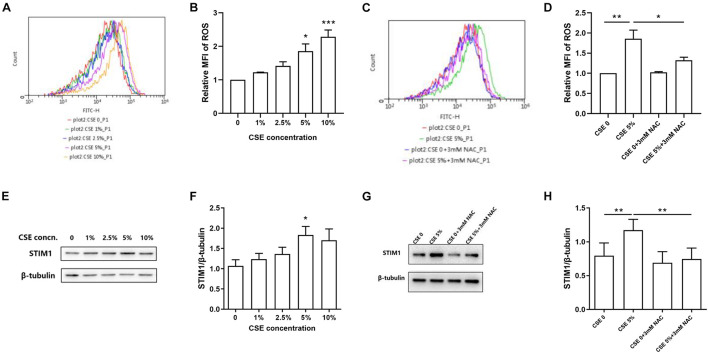
N-acetylcysteine (NAC) alleviates cigarette smoke extract (CSE)-induced ROS production and STIM1 expression in PMA-differentiated THP-1 cells. The representative images of flow cytometry for intracellular ROS levels are shown **(A,C)** and quantified using FlowJo **(B,D)**. The intracellular level of ROS was up-regulated as CSE concentration increased in PMA-differentiated THP-1 cells. Pretreatment with 3 mM NAC for 1 h alleviated CSE-induced ROS increase in PMA-differentiated THP-1 cells. The representative images of western blot of STIM1 expression are shown **(E,G)** and quantified using ImageJ **(F,H)**. 5%CSE stimulation for 48 h enhanced STIM1 expression in PMA-differentiated THP-1 cells. Pretreatment with 3 mM NAC for 1 h alleviated CSE-induced STIM1 increase in PMA-differentiated THP-1 cells. Data are displayed as mean ± SEM of at least three independent experiments. *P*-values were calculated using one-way ANOVA followed by Newman–Keuls test. *P < 0.05, **P < 0.01, and ***P < 0.001 represent significant differences. MFI, Mean fluorescent intensity; ROS, reactive oxygen species; CSE, cigarette smoke extract; NAC, N-acetylcysteine; STIM1, stromal interaction molecule 1; and PMA, phorbol myristate acetate.

Cell viability was not affected by CSE at a concentration lower than or equal to 10% (Supplementary Figure 3). To assess the potential effects of CSE and NAC on intracellular ROS production, PMA-differentiated THP-1 cells were incubated with or without CSE at different concentrations for 3 h. Cells were also pretreated with 3 mM NAC or control medium for 1 h before CSE treatment. Flow cytometry analysis showed that 5 and 10% CSE significantly induced ROS production in cells (Figures 3A,B). CSE at a concentration of 5% was used to investigate the effect of NAC on ROS production. Compared with the control groups, cells treated with 5% CSE showed increased intracellular ROS production, while pretreatment with 3 mM NAC markedly reduced CSE-induced ROS production (Figures 3C,D).

To further examine whether the expression of STIM1 was regulated by CSE-induced ROS, we measured the expression of STIM1 in PMA-differentiated THP-1 cells treated with different concentrations of CSE. We found that 5% CSE significantly increased the expression of STIM1 (Figures 3E,F). However, 1-h pretreatment with 3 mM NAC markedly suppressed CSE-induced upregulation of STIM1 in cells (Figures 3G,H). These results support the hypothesis that CSE-induced ROS may be involved in the regulation of STIM1 expression in macrophages.

### Interleukin-8 Induction Is Reduced by N-Acetylcysteine and Stromal Interaction Molecule 1 Knockdown in Cigarette Smoke Extract-Stimulated Phorbol Myristate Acetate-Differentiated THP-1 Cells

**FIGURE 4:**
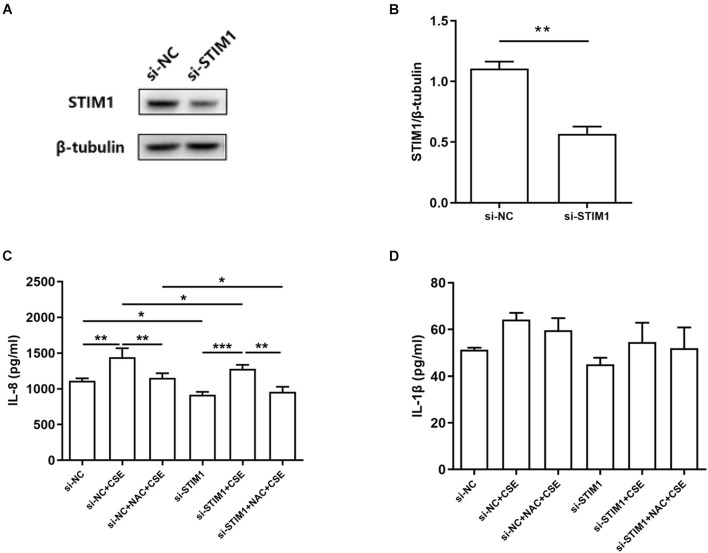
Interleukin-8 (IL-8) induction is mitigated by NAC and STIM1 knockdown in CSE-stimulated PMA-differentiated THP-1 cells. Relative STIM1 expression was detected by western blot in PMA-differentiated THP-1 cells after transfected with small interfering RNA (siRNA)-STIM1 **(A)** and quantified using ImageJ **(B)**. *P*-values were calculated using Student’s *t*-test of at least three independent experiments. si-NC transfected and si-STIM1 transfected THP-1 cells were pretreated with 3 mM NAC for 1 h, and then the supernatant was collected 48 h after 5% CSE stimulation. The levels of IL-8 and IL-1β released from the cells are shown **(C,D)**. Data are expressed as mean ± SEM of at least three independent experiments. *P*-values were calculated using Student *t*-test or one-way ANOVA followed by Newman–Keuls test as appropriate. *P < 0.05, **P < 0.01, and ***P < 0.001 represent significant differences. NC, negative control; STIM1, stromal interaction molecule 1; NAC, N-acetylcysteine; CSE, cigarette smoke extract; and PMA, phorbol myristate acetate.

The above data showed that intracellular ROS levels were upregulated by CSE treatment and NAC alleviated CSE-induced ROS production. CSE-induced upregulation of STIM1 in PMA-differentiated THP-1 cells was also inhibited by NAC. Thus, we speculated that CSE might increase the expression of STIM1 by promoting the production of ROS. To further investigate the effects of CSE-induced ROS on inflammatory cytokines and to determine whether it was subjected to the regulation of STIM1, we examined the effects of *STIM1* knockdown and NAC pretreatment on IL-8 and IL-1β, two important cytokines in the pathogenesis of COPD, in PMA-differentiated THP-1 cells (Pauwels et al., 2011). Western blot was used to confirm *STIM1* knockdown in cells (Figures 4A,B). NAC pretreatment suppressed CSE-induced IL-8 release in cells transfected with si-NC or si-STIM1. Knockdown of *STIM1* further decreased the level of IL-8 in CSE-stimulated cells (Figure 4C). These results indicated a potential regulation of IL-8 production by ROS and STIM1 in CSE-challenged cells. Combined with previous findings that ROS regulated the expression of STIM1, we hypothesized that the regulation of IL-8 by ROS and STIM1 was initiated by CSE. Neither *STIM1* knockdown nor NAC pretreatment significantly altered the production of IL-1β (Figure 4D).

### Reactive Oxygen Species/Stromal Interaction Molecule 1 Regulates Interleukin-8 Production Through Intracellular Ca^2+^ Levels in Cigarette Smoke Extract-Stimulated Phorbol Myristate Acetate-Differentiated THP-1 Cells

**FIGURE 5:**
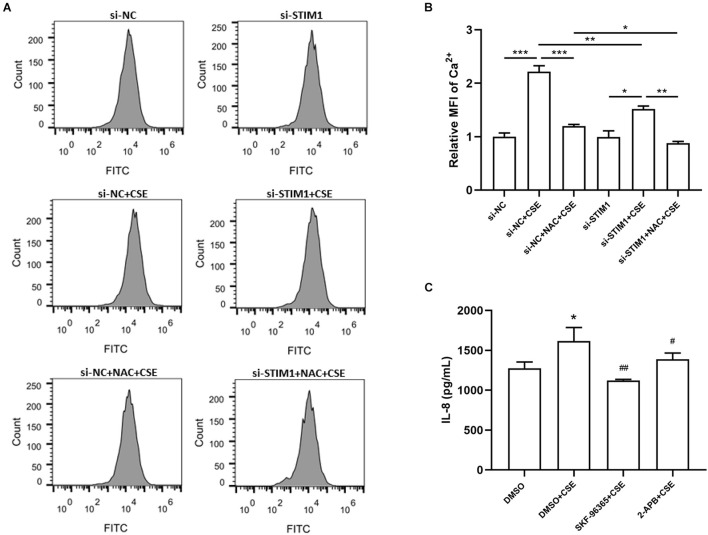
Reactive oxygen species-stromal interaction molecule 1-regulated IL-8 increase is mediated by intracellular Ca2^+^ level in CSE-stimulated PMA-differentiated THP-1 cells. The representative images of flow cytometry for detection of intracellular Ca2 + are shown **(A)** and quantified by FlowJo **(B)**. Knockdown of STIM1 and pretreatment of 3 mM NAC in PMA-differentiated THP-1 cells alleviated CSE-induced intracellular Ca2 + increase. 2-h Pretreatments by SKF-96365 and 2-APB, respectively, the Ca2 + channel inhibitors, mitigated IL-8 increase 48 h after 5%CSE stimulation **(C)**. Data are expressed as mean ± SEM of at least three independent experiments. *P*-values were calculated using one-way ANOVA followed by Newman–Keuls test. *P < 0.05, **P < 0.01, and ***P < 0.001 vs. si-NC or DMSO. ^#^*P* < 0.05, ^##^*P* < 0.01 vs. DMSO + CSE. NC, negative control; STIM1, stromal interaction molecule 1; MFI, Mean fluorescent intensity; CSE, cigarette smoke extract; NAC, N-acetylcysteine; DMSO, dimethyl sulfoxide; and PMA, phorbol myristate acetate.

To study whether intracellular Ca^2+^ is involved in the regulation of IL-8 by ROS/STIM1 in CSE-stimulated cells, we performed flow cytometry to detect intracellular Ca^2+^ levels. The results showed that pretreatment with NAC suppressed CSE-induced increase in intracellular Ca^2+^ levels, and *STIM1* knockdown further decreased intracellular Ca^2+^ levels in CSE-stimulated cells (Figures 5A,B). Moreover, treatment with SKF-96365 and 2-APB, two pharmacological inhibitors targeting Ca^2+^ channels, significantly inhibited CSE-induced increase in IL-8 levels (Figure 5C). Taken together, it could be concluded that CSE promoted the release of IL-8 in PMA-differentiated THP-1 cells possibly through the ROS/STIM1/Ca^2+^ axis.

## Discussion

**FIGURE 6:**
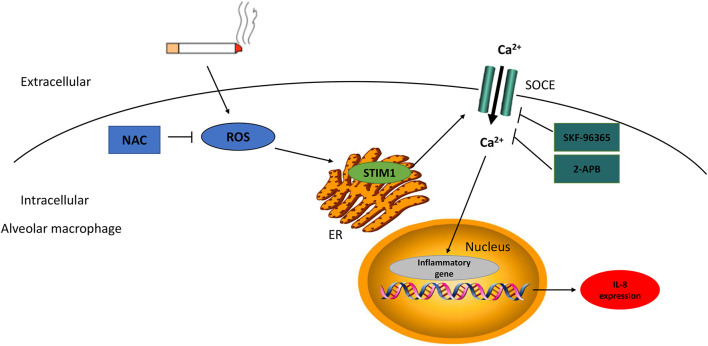
The scheme diagram of STIM1 in the regulation of cigarette smoke (CS)-stimulated alveolar macrophage. CS-induced intracellular ROS production enhanced STIM1 expression in alveolar macrophage, and further promoted inflammatory cytokine secretion through the regulation of Ca2 + entry. NAC can alleviate intracellular ROS levels and SKF-96365/2-APB can dampen Ca2 + influx, both of which can mitigate the IL-8 expression. NAC, N-acetylcysteine; ROS, reactive oxygen species; STIM1, stromal interaction molecule 1; SOCE, store-operated Ca2 + entry; and ER, endoplasmic reticulum.

Although the major characteristic of COPD, oxidative stress and pulmonary inflammation, have been reported extensively (McGuinness and Sapey, 2017; Lange et al., 2021), the link between the two pathological processes remains poorly understood. The current study investigated the potential interplay between ROS production and inflammatory cytokine expression, as well as the role of STIM1, in alveolar macrophages in response to CS. We first demonstrated that the expression level of STIM1 in the lung tissue homogenates and alveolar macrophages isolated from the BALF of COPD patients was significantly higher than that of HN and HS. Also, CSE upregulated the expression of STIM1 *in vitro*. Moreover, intrapulmonary ROS production was markedly enhanced in COPD patients. Correlation analysis indicated a possible link between ROS production and STIM1 expression. In macrophages differentiated from THP-1 cells, administration of NAC, an inhibitor of ROS, effectively suppressed CSE-induced upregulation of STIM1, implying the regulation of STIM1 by ROS in response to CS. Importantly, both NAC pretreatment and *STIM1* knockdown inhibited CSE-induced increase in intracellular Ca^2+^ levels and IL-8 release. Collectively, our study uncovered that CS promoted IL-8 secretion in human alveolar macrophages potentially through the ROS/STIM1/Ca^2+^ axis (Figure 6). These findings may contribute to a better understanding of the pathogenesis of COPD in terms of oxidative stress and inflammation.

Cigarette smoke contains approximately 4,700 chemical compounds and is implicated in many diseases, including cancer, cardiovascular diseases, and COPD (Stedman, 1968). Previous studies have demonstrated that CS increases ROS production in lung tissues and BALF both *in vivo* (Toledo et al., 2012; Li et al., 2013) and *in vitro* (Kayyali et al., 2003; Cipollina et al., 2014; Campos et al., 2017; Li et al., 2018). It has also been reported that CSE may provoke ROS production by enhancing the transcription and activity of ROS-generating enzymes (Kayyali et al., 2003). ROS may promote inflammation by activating stress kinases (e.g., c-Jun activated kinase, extracellular signal-regulated kinase, and p38) and redox-sensitive transcription factors (e.g., NF-κB and activator protein-1), which further induce the expression of multiple inflammatory genes, such as IL-8 and TNF-α (Rahman and Adcock, 2006; Zuo and Wijegunawardana, 2021). ROS also affect Ca^2+^ homeostasis by regulating Ca^2+^ transport proteins located in the plasma membrane, ER, and mitochondria. The link between ROS and inflammation in the lung has been explored (Madreiter-Sokolowski et al., 2020). Lin et al. (2015) found that CSE increased the levels of ROS in lung epithelial cells, which activated transient receptor potential ankyrin 1, increased Ca^2+^ influx, activated the MAPKs/NF-κB signaling pathway, and ultimately induced the expression of IL-8. Intracellular Ca^2+^ response can be completed inhibited by NAC (Lin et al., 2015). Previous studies have identified STIM1 as an important oxidative stress sensor (Hawkins et al., 2010) and an ER transmembrane protein (Soboloff et al., 2012), which senses the reduction of Ca^2+^ in the ER and promote inflammatory responses (Liou et al., 2005; Chen et al., 2013). That is to say, STIM1 translocates to the regions of the ER close to the plasma membrane, where it couples with and activates the plasma membrane Ca^2+^ channel protein Orai1 to increase Ca^2+^ influx (Park et al., 2009).

In this study, we found that STIM1 was mainly located in the cytoplasm of alveolar macrophages and the level of STIM1 in the lung tissues and alveolar macrophages of COPD patients was upregulated compared to other groups, which was consistent with the functional characteristics of STIM1. Moreover, correlation analysis demonstrated a significant negative association between STIM1 expression and lung function, indicating that STIM1 plays a vital role in the development of COPD. We further demonstrated that CSE upregulated STIM1 in human alveolar macrophages isolated from BALF and PMA-differentiated THP-1 macrophages *in vitro*. STIM1, as a critical oxidative stress sensor, is regulated by ROS through residue modification (Hawkins et al., 2010; Gandhirajan et al., 2013). However, whether ROS would affect the protein expression of STIM1 has not been reported. Here, we reported that NAC, a ROS inhibitor, suppressed CSE-induced upregulation of STIM1 protein in macrophages, suggesting the regulation of STIM1 by ROS.

Changes in intracellular Ca^2+^ levels are essential for proinflammatory cascade in macrophages, the predominant type of inflammatory cells in COPD (Zhou et al., 2006). Elevated IL-8 and IL-1β levels have been observed in the BALF and lung tissues of smokers and patients with COPD (Culpitt et al., 2003; Tomaki et al., 2007). Previous studies revealed that STIM1 played a role in IL-8 production in response to various stimuli in different types of cells (Zhou et al., 2014; Hong et al., 2015). In the current study, we speculated that CSE, a main resource of ROS, might promote Ca^2+^ influx by regulating STIM1 (a Ca^2+^ sensor) and further increase the production of IL-1β and IL-8 in human alveolar macrophages. Here, we unexpectedly found that CSE enhanced intracellular Ca^2+^ levels, but this effect was alleviated by *STIM1* knockdown and NAC pretreatment, suggesting the regulation of intracellular Ca^2+^ levels by ROS and STIM1. Consistent with previous studies (Sarir et al., 2010), NAC inhibited CSE-induced upregulation of IL-8 in human macrophages. Knockdown of *STIM1* by siRNA also decreased CSE-induced IL-8 release. The expression of IL-1β, however, was not affected by NAC pretreatment or *STIM1* knockdown. Subsequently, we treated cells with Ca^2+^ channel inhibitors (SKF-96365 and 2-APB) before CSE stimulation to explore the role of intracellular Ca^2+^ on the expression of inflammatory cytokine IL-8. The results showed that Ca^2+^ channel inhibitors suppressed CSE-induced upregulation of IL-8, indicating that Ca^2+^ plays a critical role in CSE-stimulated inflammatory responses. These findings imply that STIM1 may regulate oxidative-stress-related inflammation by acting as a link between ROS and inflammatory cytokines, such as IL-8.

The present study has some limitations. Firstly, we did not validate the role of the ROS/STIM1/Ca^2+^ axis in COPD in genetic knockout animal models. In addition, further studies are needed to elucidate the exact mechanisms of ROS-mediated upregulation of STIM1 in CSE-stimulated human alveolar macrophages.

## Conclusion

In conclusion, our study demonstrated that STIM1 was mainly expressed in alveolar macrophages and the level of STIM1 was increased in patients with COPD. Mechanically, CSE promoted the expression of inflammatory cytokine IL-8 in PMA-differentiated THP-1 macrophages possibly through the ROS/STIM1/Ca^2+^ axis. These findings revealed a critical role of STIM1 in ROS-related pulmonary inflammation in COPD and provided new insights into the pathogenic mechanism of COPD.

## Data Availability Statement

The raw data supporting the conclusions of this article will be made available by the authors, without undue reservation.

## Ethics Statement

The studies involving human participants were reviewed and approved by the Tongji Hospital Ethics Committees. The patients/participants provided their written informed consent to participate in this study.

## Author Contributions

JX: conceptualization, methodology, supervision, and funding acquisition. XZ and YZ: project administration, investigation, formal analysis, software, and writing – original draft. YG and ZD: investigation. QH and TW: software. All authors contributed to the article and approved the submitted version.

## Conflict of Interest

The authors declare that the research was conducted in the absence of any commercial or financial relationships that could be construed as a potential conflict of interest.

## Publisher’s Note

All claims expressed in this article are solely those of the authors and do not necessarily represent those of their affiliated organizations, or those of the publisher, the editors and the reviewers. Any product that may be evaluated in this article, or claim that may be made by its manufacturer, is not guaranteed or endorsed by the publisher.

## Supplementary Material

Supplementary Figure 1 Intrapulmonary ROS levels are positively associated with STIM1 expression. The correlation between ROS levels and STIM1 mRNA expression in the lung specimens of all patients are shown **(A)**. The correlation between ROS levels and STIM1 protein expression are shown **(B)**. *n* = 12 for HN, *n* = 12 for HS, and *n* = 16 for SC. STIM1, stromal interaction molecule 1; ROS, reactive oxygen species; MFI, Mean fluorescent intensity; HN, healthy non-smoker; HS, healthy smoker; SC, smoker with COPD.

Supplementary Figure 2 Cigarette smoke extract stimulation increased STIM1 expression in human macrophages isolated from BALF. Representative western blot image regarding STIM1 protein expression in macrophages treated with or without 5% CSE is shown **(A)** and quantified using ImageJ **(B)**. Data are expressed as mean ± SEM of at least three independent experiments. *P*-values were calculated using Student *t*-test. ^∗∗∗^*P* < 0.001 represents significantly different relative to control. CSE concn, concentration of cigarette smoke extract; STIM1, stromal interaction molecule 1.

Supplementary Figure 3 Cigarette smoke extract stimulation lower than 10% conducted no distinct influence on cell viability in PMA-differentiated THP-1 macrophage. The effects of CSE on HBE cell activity was shown above. Data are expressed as mean ± SEM of at least three independent experiments. *P*-values were calculated using one-way ANOVA followed by Newman–Keuls test. ^∗∗^*P* < 0.01 represents significant difference relative to control. CSE, cigarette smoke extract.

## Abbreviations

BALF: bronchoalveolar lavage fluid
Ca^2+^: calcium
COPD: chronic obstructive pulmonary disease
CRAC: Ca^2+^ release-activated Ca^2+^
CSE: cigarette smoke extract
ER: endoplasmic reticulum
IL-8: interleukin-8
IL-1 β: interleukin-1 β
NAC: N-acetylcysteine
NF- κ B: nuclear factor kappa-light-chain-enhancer of activated B cell
Nrf2: nuclear factor erythroid 2-related factor2
PMA: phorbol myristate acetate
ROS: reactive oxygen species
STIM: stromal interaction molecule
SOCE: store-operated Ca^2+^-entry.
